# Routine Computer Tomography Imaging for the Detection of Recurrences in High-Risk Melanoma Patients

**DOI:** 10.1245/s10434-017-5768-8

**Published:** 2017-01-31

**Authors:** Tristen S. Park, Giao Q. Phan, James C. Yang, Udai Kammula, Marybeth S. Hughes, Kasia Trebska-McGowan, Kathleen E. Morton, Donald E. White, Steven A. Rosenberg, Richard M. Sherry

**Affiliations:** Surgery Branch, Center for Cancer Research, National Cancer Institute, National Institutes of Health, Bethesda, MD USA

## Abstract

**Background:**

The use of routine CT imaging for surveillance in asymptomatic patients with cutaneous melanoma is controversial. We report our experience using a surveillance strategy that included CT imaging for a cohort of patients with high-risk melanoma.

**Methods:**

A total of 466 patients with high-risk cutaneous melanoma enrolled in adjuvant immunotherapy trials were followed for tumor progression by physical examination, labs, and CT imaging as defined by protocol. Evaluations were obtained at least every 6 months for year 1, every 6 months for year 2, and then annually for the remainder of the 5-year study. Time to tumor progression, sites of recurrence, and the method of relapse detection were identified.

**Results:**

The patient cohort consisted of 115 stage II patients, 328 stage III patients, and 23 patients with resected stage IV melanoma. The medium time to progression for the 225 patients who developed tumor progression was 7 months. Tumor progression was detected by patients, physician examination or routine labs, or by CT imaging alone in 27, 14, and 59% of cases respectively. Melanoma recurrences were noted to be locoregional in 36% of cases and systemic in 64% of cases. Thirty percent of patients with locoregional relapse and 75% of patients with systemic relapse were detected solely by CT imaging.

**Conclusions:**

CT imaging alone detected the majority of sites of disease progression in our patients with high-risk cutaneous melanoma. This disease was not heralded by symptoms, physical examination, or blood work. Although the benefit of the early detection of advanced melanoma is unknown, this experience is relevant because of the rapid development and availability of potentially curative immunotherapies.

Although the risk for relapse for patients with stage II, III, and resected stage IV cutaneous melanoma is recognized to be high, optimal follow-up surveillance strategies remain controversial. The wide variation in surveillance practice patterns is unfortunate but understandable given the lack of level 1 evidence, conflicting retrospective data, and the significant cost associated with aggressive follow-up. The National Comprehensive Cancer Network (NCCN) follow-up guidelines for stage IIB or greater melanoma recommend physical examination every 3 to 6 months with chest x-ray and serum LDH and to consider annual computed tomography (CT) scans.^1^ This report describes the results of our experience utilizing an aggressive surveillance strategy that was strictly defined and implemented as a component of adjuvant immunotherapy protocols conducted for patients with melanoma at the Surgery Branch, NCI.

## Methods

### Patient Selection

A retrospective analysis was performed using patients enrolled in one of four different institutional review board-approved adjuvant immunotherapy trials conducted in the Surgery Branch, National Cancer Institute between 1998 and 2009. The inclusion criteria for these four studies were identical and included patients with stage II, stage III, and resected stage IV cutaneous melanoma. Patients with ulcerated or ≥1.5-mm primary melanomas, completely resected local regional nodal disease, or completely resected metastatic disease were eligible if HLA appropriate and enrolled within 6 months of surgery. These trials were designed using the 5^th^ edition of the AJCC staging for cutaneous melanoma as recognized in 1998. Staging was modified to include tumor ulceration.^2^,^3^ Patients with ocular or mucosal melanoma or who required steroids were excluded. Eligible patients were screened with physical exam, lab tests, brain MRI, and CT scan of chest, abdomen, and pelvis. Following adjuvant immunotherapy, patients were monitored closely for recurrence by physical examination, labs, and imaging as required by protocol for 5 years. Patient follow-up was censored for disease progression or discharge from clinic at year 5. Some patients elected to continue annual surveillance at the Surgery Branch off protocol beyond 5 years, although data generated beyond 5 years was not included in this report.

### Protocols and Surveillance Strategy

The Surgery Branch, NCI conducted three successive pilot adjuvant immunotherapy protocols to evaluate peptide immunization to a variety of melanocyte differentiation antigens, including gp 100, MART-1, and tyrosinase. The fourth protocol was a cellular vaccine consisting of peripheral blood lymphocytes transduced with a T-cell receptor (TCR) that recognized the HLA-A*02-restricted MART-1 antigen. Details of these studies can be found by using the Clinical trials.gov identifier noted in Table 1. All protocols required CT imaging of chest, abdomen, and pelvis and MRI brain imaging within 4 weeks of protocol enrollment. Subsequent brain imaging was obtained if neurologic symptoms were detected or as part of a metastatic survey following disease progression at other sites. Because each protocol had a different vaccination schema, there were minor variations in surveillance schedules during year 1. However, all patients had complete clinical evaluations and CT imaging within 4 weeks of protocol enrollment and at least two more times during the first year of the study. Subsequent clinical and imaging evaluations were very similar for all four of the clinical trials. The protocol surveillance schedules are described below and outline in Table 2. The results of these pilot studies have been published in part.^4^^–^ 6

**Table 1:** Clinical and pathological characteristics of patients with initial diagnosis of AJCC stage II–IV melanoma

Age (year)	
Median	49
Range	17–79
Gender	No. of patients (%)
Male	295 (63)
Female	171 (37)
Primary site	
Head and neck	68 (15)
Extremities	193 (41)
Trunk	167 (36)
Other	38 (8)
Stage	
II	115 (25)
III	328 (70)
IV	23 (5)
Adjuvant therapy	
00C0216 (Tyr 240-251/IF or gp100	204 (44)
03C0172 (MART 1:27-35)	113 (24)
06C0069 (gp100:209-217M/montar)	101 (22)
08C0162 (MART F5TCR +ALVAC)	48 (10)
Total	466

Total enrolled: 491

Total evaluable: 466

Not evaluable: 25

**Table 2:** Vaccine protocol surveillance schedule

	Treatment phase/year 1 clinical evaluation	Treatment phase/year 1 CT imaging	Year 2 Clinic/with CT	Years 3–5 Clinic/with CT
NCT 00020358 (N=204)	Every 3 months	Every 3 months	Every 6 months	Yearly
NCT 00059475 (N = 113)	Every 3 months	Every 6 months	Every 6 months	Yearly
NCT 00273910 (N = 101)	Every 3 and 9 months	Every 3 and 9 months	Every 6 months^a^	Yearly
NCT 00706992 (N = 48)	Every 3, 6, 12 months	Every 3, 6, 12 months	Every 6 months	Every 6 months

^a^A small subset of this cohort elected to start annual surveillance at 18 months

## Recurrence Classification

Patients documented to have progressive melanoma were analyzed for demographic information, time and site of first recurrence, and the method of detection of tumor progression (patient detection, physician examination/lab abnormality, or CT imaging). For CT imaging to have been considered the method that detected disease progression, patients were required to be asymptomatic and to have normal labs and physical examination. For physician examination/lab abnormality to have been considered the method of detection, patients also were required to be asymptomatic. Finally, patients who presented with symptoms or new findings detected by the patient were categorized as patient-detected. For this group, no distinction was made between patients who had tumor recurrence identified at a regularly scheduled follow-up visit and those who an evaluation for symptoms more urgently at an unscheduled appointment.

Locoregional relapse was defined as the identification new local/intransit or regional nodal. Systemic relapse included identification of intrathoracic, bone, intra-abdominal and CNS, as well as cutaneous/muscle/nodal recurrences, which were distant from the primary site.^7^ Patients who relapsed at several sites concomitantly were scored on the basis of the site that was most advanced.

## Results

### Patient Demographics and Relapse Incidence

A total of 466 patients enrolled on an adjuvant immunotherapy trials were identified and evaluated. Of these, 115 (25%) had resected stage II disease, 328 (70%) had stage III, and 23 (5%) had stage IV disease. Sixty-three percent of patients were male, and 37% were female. The median patient age was 49 years. The majority of patients had a primary located on an extremity (41%), 36% had a primary located on the trunk, 15% had a head or neck primary, and 8% had a primary of unknown origin (Table 1).

**Fig. 1:**
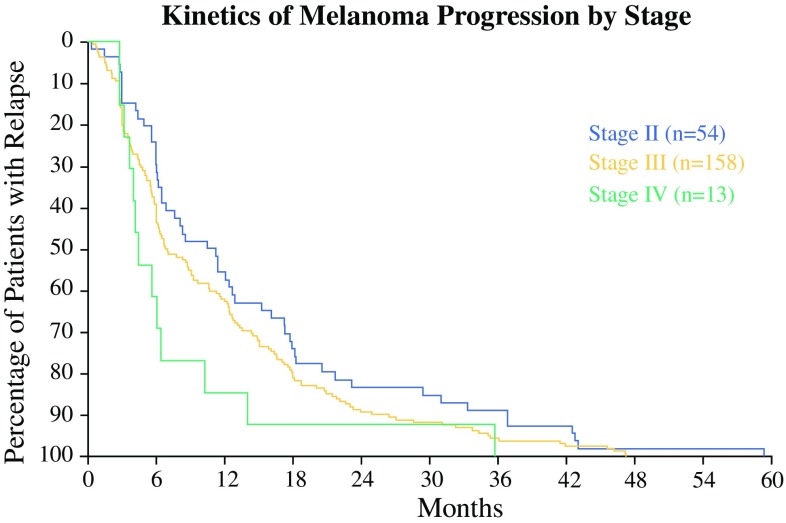
Kinetics of melanoma progression by stage. Relapse-free survival of all 225 patients with melanomas stage II, III, IV who relapsed

Of the 466 patients, 225 (48%) developed disease progression during the 5-year observation period. For these patients, the median time from protocol enrollment to the date of identified tumor progression was 7 months. There were no differences in the rates or timing of relapses between the four adjuvant trials. Approximately 94% of relapses were detected by 3 years and 99% of relapses were noted by 4 years. Not surprisingly, stage IV patients were more likely to demonstrate early disease progression (Fig. 1).

### Site of First Relapse

Table 3 documents the sites of disease progression as either local regional or systemic using the previously defined criteria for the 225 patients who developed relapse. The first relapse among stage II patients was locoregional in 52% of patients and systemic in 48% of patients. Among those with stage III disease, the site of tumor progression was locoregional in 32% of patients and systemic in 68% of patients. For patients with resected stage IV disease at protocol enrollment and who demonstrated tumor progression, the disease was identified as locoregional in 23% of patients and systemic in 77% of patients.

**Table 3:** Site of first recurrence

Stage	Total patients	Total recurrence	Locoregional	Systemic
Stage II	115	54	28 (52%)	26(48%)
Stage III	328	158	50 (32%)	108 (68%)
Stage IV	23	13	3 (23%)	10 (77%)
Total	466	225	81 (36%)	144 (64%)

Locoregional is defined as local, in-transit, and nodal metastases

### Method of Detection

The method by which disease progression was first detected was classified as by patient, by physician or lab analysis, or by CT imaging alone (Table 4). For the 225 individuals who recurred, 60 were detected by the patient (27%), 32 by physicians or labs (14%), and 131 by CT imaging alone (59%). For the 81 patients with local regional recurrence, 36 (45%) recurrences were patient-detected, 20 (25%) were detected by physician examination, and 24 (30%) were identified by imaging alone. Not surprisingly, patients and physicians were more likely to diagnose locoregional disease and less likely to detect progressive systemic disease.

**Table 4:** Method of detection by site and stage

Total recurrences	Patient detected^a^	Physician detected^a^	Imaging detected
225	60 (27%)	32 (14%)	131 (59%)
By site			
Locoregional—81	36 (45%)	20 (25%)	24 (30%)
Systemic—144	24 (17%)	12 (8%)	107 (75%)
By stage			
II—54	18 (33%)	6 (11%)	30 (56%)
III—158	37 (23%)	26 (17%)	95 (60%)
IV—13	5 (38%)	0	6 (46%)

^a^Two patients were detected by physical exam but it was unclear whether by patient or physician exam

*Pt 1* presented as stage 4, recurred systemically; *Pt 2* presented as stage 4, recurred locoregionally

As expected, CT imaging was the most effective method to detect systemic progression of melanoma. For the 144 patients who developed systemic recurrence, 107 (75%) were detected by imaging in an asymptomatic patient with normal labs and physical examination. Twenty-four (17%) recurrences were patient-detected. These individuals noted a new tumor and had concomitant metastatic disease detected or complained of pain, bleeding, or neurologic symptoms. Nine patients had their systemic disease detected by physician examination, and only three patients had abnormal laboratory finding which suggested disease progression. These individuals developed anemia from intestinal metastasis and are included in the physician-detected cohort. As noted in Table 4, two patients had metastatic disease detected by physical examination, but the medical record was unclear if this was by the patient’s or physician’s examination. Both of these patients were in the resected Stage IV cohort; one recurred with locoregional disease and one with systemic disease.

Serum LDH was routinely obtained at each follow-up evaluation. LDH elevation was recorded at least once in 13% of patients who remained free of disease during the observation period and in only 3.5% of patients at the time tumor progression was detected in patients with tumor progression. Neither LDH nor LFTs appeared to be useful for surveillance.

### Detection of Brain Metastasis

Melanoma brain metastases were detected in 17 (4%) of 466 evaluable patients and in 7.5% of the 225 patients who developed progressive melanoma during surveillance. Given our brain surveillance strategy, it is not surprising that 14 of these patients presented with neurologic symptoms, including headache, visual changes, or seizures. The remaining three patients had brain disease identified as part of a complete metastatic survey that was initiated, because progression had been detected at other sites. Interestingly, 11 of the 17 patients with brain metastasis patients had brain-only disease. Sixteen of the 17 patients had stage III or resected stage IV disease at protocol entry.

## Discussion

This report describes the results of a surveillance strategy for patients with stage II, III, and resected stage IV cutaneous melanoma that was implemented for adjuvant immunotherapy trials conducted at the Surgery Branch, NCI, and that included the routine use of CT imaging. Although there was some variation in the timing of evaluations during the first year for each protocol, surveillance for all patients included imaging of the chest abdomen and pelvis at intervals of 6 months or less for year 1, every 6 months for year 2, and then annually through year 5 and protocol termination. We found that CT imaging identified a large number of cases of progressive melanoma that were missed by both patients and physicians. This was evident whether the site of disease progression was systemic or locoregional. This also was the case for our patients regardless of their stage at time of protocol enrollment.

Current NCCN guidelines for surveillance of patients with high-risk melanoma consider routine cross-sectional imaging to be optional. Reports that have addressed this issue generally have used CT imaging to evaluate patient symptoms or physical findings.^8^^–^ 13 One notable exception is the report by Romero et al. from Memorial Sloan-Kettering Cancer Center.^7^ In this retrospective series, patients with stage III melanoma were evaluated every 3 months for 2 years and subsequently every 6 months. Although the imaging frequency was not specified, CT images were said to be obtained typically before each evaluation. These authors noted that 53% of tumor relapses were detected only by CT imaging. A fact that also was consistent with our findings was that serum LDH and LFTs were not helpful when screening patients at risk for melanoma recurrence. However, in our series we did detect gastrointestinal metastasis as an initial site of disease progression in three patients following an evaluation performed solely to evaluate asymptomatic anemia.

Our experience confirms that a surveillance strategy for asymptomatic patients with high-risk cutaneous melanoma should include routine cross-sectional imaging. CT surveillance identified a large number of locoregional relapses missed by patients and physicians as well as unsuspected systemic disease. Of the 225 patients who recurred during the 5-year observation period, 13 (6%) developed tumor progression after year 3. Although NCCN guidelines do not recommend imaging beyond 3 years, 11 of these 13 patients had disease progression detected by imaging alone. Our future adjuvant immunotherapy protocols will include CT imaging every 6 months for the first 2 years followed by annual imaging. Blood work will include a CBC. Obviously, our report does not help to clarify surveillance techniques beyond 5 years. Finally, PET imaging was obtained on only 16 patients in this series. Consequently, our experience does not address the role of PET in surveillance strategies for patients at high risk for developing progressive melanoma.

We recognize that surveillance for patients at risk for melanoma relapse has not been documented to improve overall survival. However, the recent approval of adjuvant ipilimumab obviously has changed the therapeutic landscape for these patients and highlights the need for effective surveillance.^14^ The development of multiple and potentially curative immunotherapies, including high-dose interleukin-2, check point inhibitors, and ACT, make the timely identification of metastatic melanoma a high priority.^15^–^21^
